# Body Composition Predictors of Complicated Crohn's Disease

**DOI:** 10.1159/000529426

**Published:** 2023-01-31

**Authors:** Felix Barajas Ordonez, Bohdan Melekh, Pablo Rodríguez-Feria, Oksana Melekh, Maximilian Thormann, Robert Damm, Jazan Omari, Maciej Pech, Alexey Surov

**Affiliations:** ^a^University Clinic for Radiology and Nuclear Medicine, University Hospital Magdeburg, Magdeburg, Germany; ^b^Department of International Health, Care and Public Health Research Institute, Maastricht University, Maastricht, The Netherlands; ^c^Radiology Practice, Dessau, Germany; ^d^Department of Radiology, Neuroradiology and Nuclear Medicine, Johannes Wesling University Hospital, Ruhr University Bochum, Minden, Germany

**Keywords:** Crohn's disease, Fistula, Subcutaneous adipose tissue, Visceral adipose tissue, Sarcopenia

## Abstract

**Background:**

High visceral adipose tissue (VAT) and creeping fat (CrF) in Crohn's disease (CD) have been widely recognized. The VAT to subcutaneous adipose tissue (SAT) ratio and sarcopenia have been associated with CD complications. Studies regarding the influence of body composition predictors on CD complications assessed with magnetic resonance enterography (MRE) are scarce.

**Aim:**

The aim of this study was to assess body composition parameters and CrF in opportunistic MRE as predictors of complicated CD.

**Methods:**

This was a retrospective study of 114 patients with inflammatory (*n* = 54) and complicated (*n* = 60) CD. The semiautomated assessment of body composition and the qualitative evaluation of CrF were performed.

**Results:**

Body composition parameters did not differ between both groups regarding the body mass index (*p* = 0.50), total adipose tissue index (TATI) (*p* = 0.14), subcutaneous adipose tissue index (SATI) (*p* = 0.17), visceral adipose tissue index (VATI) (*p* = 0.33), VAT/SAT ratio (*p* = 0.77), intramuscular adipose tissue (*p* = 0.64), skeletal muscle index (*p* = 0.22), and sarcopenia (*p* = 0.50). 47 strictures, 18 fistulae, and seven abscesses were identified. Fistulae were more likely to occur in patients with CrF (odds ratio [OR] 5.07, 95% confidence interval [CI] 1.76–14.56; *p*=<0.001) and high VAT/SAT ratio (OR: 3.82, 95% CI 1.34–10.85; *p* = 0.01).

**Conclusion:**

Body composition measurements in CD patients displayed no statistically significant difference between the groups of inflammatory and complicated disease. Nonetheless, CD patients stratified in the group of high VAT/SAT ratio and the presence of CrF should be recognized as risk groups for the occurrence of fistulae.

## Introduction

Crohn's disease (CD) is a progressive disorder characterized by recurring inflammation in the gastrointestinal tract [1]. CD behavior is dynamic over time and progression is shaped by the complications [2, 3, 4]. CD complications, including stricture, fistula, and abscess are significant events in diseases' course, leading to higher morbidity rates and impaired quality of life, and higher social and economic burden on healthcare systems [5, 6, 7].

Hypertrophy of visceral adipose tissue (VAT) and creeping fat (CrF) in CD patients have been widely recognized [8]. CrF is defined as an expansion of adipose tissue around the inflamed and fibrotic intestine [9]. Hypertrophic VAT releases higher levels of interleukins, leading to increased inflammatory response, promoting damage to the mucosa, and potentially increasing the risk of developing complications [8]. Erhayiem et al. [8], in 2011, recognized a high VAT to subcutaneous adipose tissue (SAT) ratio as a potential risk factor for stricturing and fistulizing complications in CD. Accordingly, Bryant et al. [10], in 2018, suggested the importance of the VAT/SAT ratio as a potential biomarker rather than body mass index (BMI) for stricturing complications in CD.

CD has been traditionally associated with malnutrition and lower body mass index (BMI) [11]. Both are key drivers of low skeletal muscle tissue (SMT) and the consequential loss of function, a condition known as sarcopenia [11, 12]. Grillot et al. [13], in 2020, identified that sarcopenia in CD patients negatively impacts the length of hospital stay and surgical outcomes. Zhou et al. [14], in 2021, reported an association between low SMT and complicated CD.

In CD patients magnetic resonance enterography (MRE) is an essential pillar for diagnosis, assessment of disease severity, and complications (outside the acute setting) [15]. Its advantages included the avoidance of radiation exposure and good diagnostic accuracy [16]. So far, the quantification of body composition parameters in CD has been mainly performed on computer tomography (CT) scans [4, 17, 18, 19, 20, 21, 22]. Studies regarding the influence of body composition predictors on CD complications assessed with MRE are scarce. This study aimed at retrospectively assessing body composition parameters and CrF on opportunistic MRE scans as predictors of complicated CD.

## Patients and Methods

### Setting and Participants

**Fig. 1:**
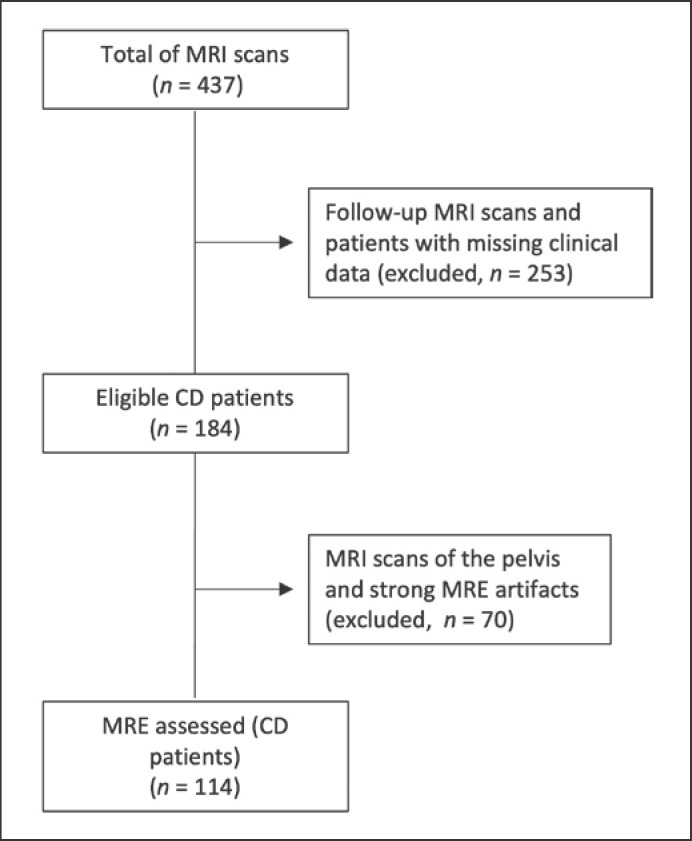
Flowchart of patient selection. CD, Crohn's disease; MRI, magnetic resonance imaging; MRE, magnetic resonance enterography.

Patients with diagnosed CD who underwent a magnetic resonance imaging (MRI) scan in the University Clinic for Radiology and Nuclear Medicine (University Hospital Magdeburg) between June 2010 and April 2020 were retrospectively assessed. MRE scans were performed to evaluate disease extension, discard complications, or evaluate therapy response. The inclusion criteria were: (1) first MRE chronologically available and (2) anthropometric data in the clinical records. The exclusion criteria were: (1) MRI of the pelvis and (2) strong MRI artifacts. 437 MRI scans were identified, of which 184 were the first MRI scans. 70 MRI scans were excluded due to examination of the pelvic region or imaging artifacts. The final population was composed of 114 MRE scans (CD patients). A flowchart of patient selection is depicted in Figure 1.

### Data Sources and Baseline Characteristics

A search of MRI scans of CD patients was conducted with the picture-archiving and communication system viewing station (INFINITT Healthcare, Seoul, South Korea). Clinical data of the identified patients were extracted from medical records using the internal database (MEDICO KIS, CompuGroup Medical SE & Co. KGaA, Koblenz, Germany). Clinical data comprised the gender, age at the baseline and at onset, height, and weight, current CD drug therapy, smoking status, and C-reactive protein (CRP) levels. The age at onset was documented based on the Montreal classification as follows: A1 (less than 16 years), A2 (between 17 and 40 years), or A3 (over 40 years) [3].

### MRE Technique

Each MRE scan was performed on a 1.5 T MRI scanner (Intera, Philips Medical Systems, Best, The Netherlands). Optimal small bowel imaging depends on adequate bowel dilatation. The MRE protocol included preparation with fasting overnight. On the day of examination, bowel dilatation was reached through oral administration of 1200 mL of 2.5% sorbitol in small aliquots over 4 h before the examination [18]. Inhibition of intestinal motility was induced by applying intravenously 20 mg/mL of N-butylscopolamine (Buscopan, Boehringer Ingelheim, Germany). A gadolinium-based MRI contrast agent (Gadovist, Bayer Vital, Leverkusen, Germany) was administrated as an intravenous bolus injection at approximately 0.1 mL/kg. The MRE sequences are displayed in online supplementary Table 1 (for all online suppl. material, see www.karger.com/doi/10.1159/000529426).

### Assessment of Complications and Creeping Fat

Each MRE scan was reviewed in tandem by two physicians to get agreement about the measurements: one senior radiology resident with 4 years of experience in the field of abdominal-pelvic MRI and a senior staff radiologist with more than 15 years of experience in the same field. For each scan, the following aspects were evaluated: (1) visible small bowel stricture, defined as a small bowel lumen <10 mm with or without prestenotic dilatation considering a prestenotic lumen >30 mm dilatation, (2) visible fistula, defined as an abnormal communication between the small bowel, and other organs, (3) visible abscess, defined as an encapsulated collection containing pus and/or gas, and (4) the presence or absence of CrF, defined as fatty deposition along the mesenteric border of inflamed bowel segment [18, 21, 23]. Depending on the MRE findings, patients were divided into inflammatory and complicated disease. Complicated disease was defined as the presence of stricture, fistula, or abscess [3, 4, 6].

### Assessment of Body Composition

**Fig. 2:**
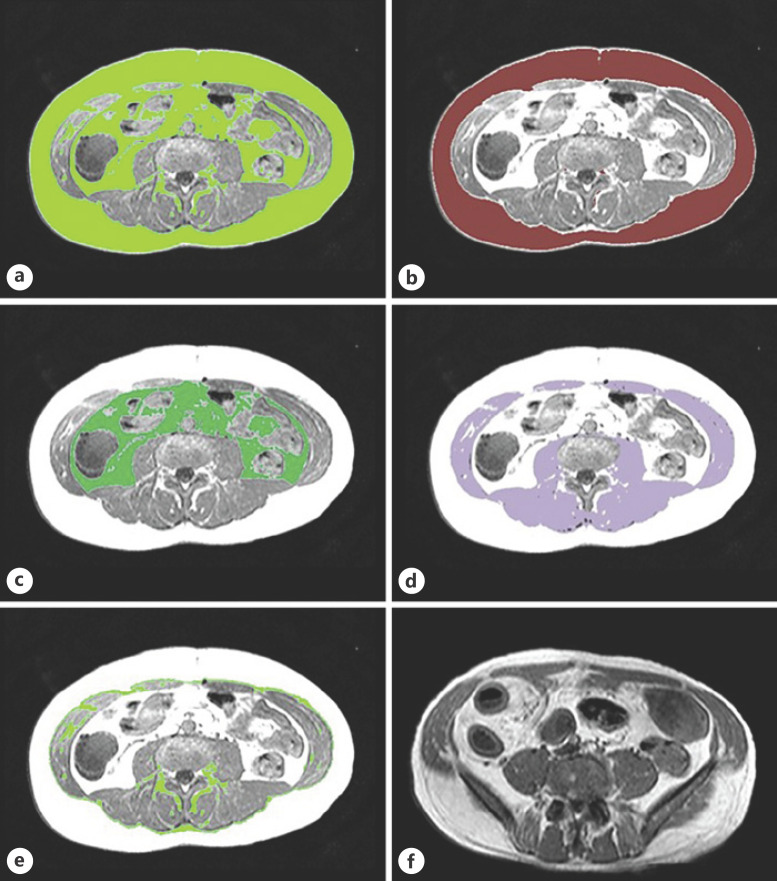
Segmentation of body composition based on cross-sectional area (CSA) measurements at the L3 inferior endplate level in MRE (**a**−**e**). **a** Total adipose tissue (TAT). **b** Subcutaneous adipose tissue (SAT). **c** Visceral adipose tissue (VAT). **d** Skeletal muscle tissue (SMT). **e** Intramuscular adipose tissue (IMAT). **f** Creeping fat (CrF), T1-weighted MRE image shows fatty deposition along the mesenteric border of inflamed bowel segment.

Body composition measurements were performed on MRE scans using the semiautomated segmentation tool AsanJ-Morphometry software (Asan Image Metrics, Seoul, Korea) [24]. The software was operated by a senior radiology resident with 4 years of experience in the field of abdominal-pelvic MRI. The cross-sectional area (CSA) measurements were evaluated at the L3 inferior endplate level. The body composition measurement at this level has often been used as a reference in clinical routine and has been the reference location for analyzing body composition [25]. It included the estimation of total adipose tissue (TAT), SAT, VAT, SMT, and intramuscular adipose tissue (IMAT) in square centimeters (cm^2^) based on the pixel count (Fig. 2). Muscle and adipose tissues were separated using thresholds for the signal intensity (SI) on precontract T1-weighted MRE scans with a value above 350 SI and lower 750 SI for adipose tissue and above 100 SI and lower 350 SI for muscle.

### Body Composition Groups

BMI was calculated by using the formula [weight (kg)/height squared (m^2^)] [26]. BMI categories were subdivided as follows: underweight (BMI <18.5 kg/m^2^), normal weight (BMI 18.5–24.9 kg/m^2^), and overweight/obese (≥25.0 kg/m^2^) [27]. Sarcopenia was measured in terms of skeletal muscle index (SMI) [12]. The SMI was calculated by dividing the SMT (cm^2^) by height squared (m^2^) [26]. The SMI cutoff values to define sarcopenia for men were 43 (cm^2^/m^2^) under a BMI of 25 (kg/m^2^) and 53 (cm^2^/m^2^) over a BMI of 25 kg/m^2^, respectively, and 41 (cm^2^/m^2^) for women [26, 28]. To calculate TATI, SATI, and VATI, TAT (cm^2^), SAT (cm^2^), and VAT (cm^2^) were divided by the height squared (m^2^), respectively. The sex-specific cutoff values for the classification of SATI (low/normal vs. high SATI) were 40 (cm^2^/m^2^) for men and 30 (cm^2^/m^2^) for women; for VATI (low/normal vs. high VATI) 44.0 (cm^2^/m^2^) for men and 35 (cm^2^/m^2^) for women; and for VAT/SAT ratio (low/normal vs. high VAT/SAT ratio) 1.08 for men and 0.86 for women [29, 30].

### Statistical Analysis

Continuous variables, including body composition parameters, are shown as mean (M) and standard deviation (SD) or median and interquartile range (IQR). The Kolmogorov-Smirnov test was used to assess the normality of the continuous variables. Continuous variables were compared between the groups of inflammatory and complicated CD using the student's *t* test. The Mann-Whitney U test was used to assess continuous, not normally distributed variables. Categorical variables, including CrF, were compared using the χ^2^ test or Fisher's exact test, as appropriate. A binary logistic regression model for body composition groups based on sex-specific values was performed to evaluate the factors associated with stricture, fistula, and abscess. Odds ratio (OR) is presented together with 95% confidence interval (CI). A two-tailed *p* value ≤0.05 was considered statistically significant. IBM SPSS Statistics for Windows, version 27.0 (IBM Corp., Armonk, NY, USA) was used as analytic software.

## Results

### Patient Characteristics

The baseline characteristics of our population (*n* = 114) are shown in Table 1. The median age at baseline was 35.50 years (IQR, 27.0–46.3). The majority of patients were male (*n* = 60, 52.6%), and the median BMI was 21.94 (kg/m^2^) (IQR, 19.4–24.9). We identified 54 patients with inflammatory and 60 with complicated CD. The groups of inflammatory and complicated disease were well-matched for gender, age at the baseline, age of onset, current drug therapy, current smoking status, BMI categories, and CRP levels (>5 ng/mL).

### Comparison of the Body Composition Parameters in the Inflammatory and Complicated Disease

Body composition parameters in the groups of inflammatory and complicated disease are listed in Table 2. There was no significant difference between both groups regarding TAT (*p* = 0.17), TATI (*p* = 0.14), SAT (*p* = 0.22), SATI (*p* = 0.17), VAT (*p* = 0.37), VATI (*p* = 0.33), VAT/SAT ratio (*p* = 0.77), IMAT (*p* = 0.64), SMT (*p* = 0.32), and SMI (*p* = 0.22).

### Comparison of Body Composition Groups Based on Sex-Specific Cutoff Values of Values, CrF, and Complications

Patients were classified into body composition groups based on sex-specific cutoff values. There were no significant differences in the occurrence of complicated disease (including all stricturing and penetrating complications) in the groups of SATI (low/normal vs. high SATI) (*p* = 0.25), VATI (low/normal vs. high VATI) (*p* = 0.84), and VAT/SAT ratio (low/normal vs. high VAT/SAT ratio) (*p* = 0.56). Sarcopenia was identified in 68 patients (59.6%) and did not differ in the groups of inflammatory and complicated disease (*p* = 0.50). CrF was identified in 29 patients. There was no significant difference in the occurrence of complicated disease if CrF was present (*p* = 0.11). Table 3 summarizes these findings.

A total of 47 strictures, seven abscesses, and 18 fistulae were identified among the patients with complicated disease. The occurrence of each complication was also assessed in each group of body composition. Only the groups with a high VAT/SAT ratio and CrF demonstrated a significant difference in the occurrence of fistulae (*p* = 0.01 and *p* = <0.001, respectively) (Table 4). None of the groups demonstrated a significant difference in the occurrence of abscesses (online suppl. Table 2a) or strictures (online suppl. Table 2b). The occurrence of CrF tended to be more common in patients with stricturing complications when compared to patients without stricturing complications (*p* = 0.18) (online suppl. Table 2b).

The association of the body composition groups, CrF, and CD complications was further explored by estimating the OR for the occurrence of fistulae (Table 5), strictures (Table 6), and abscesses (online suppl. Table 3). CrF (OR 5.07, 95% CI 1.76–14.56; *p* = <0.001) and high VAT/SAT ratio (OR: 3.82, 95%, CI 1.34–10.85; *p* = 0.01) were positively associated with the occurrence of fistulae. Neither the body composition groups nor CrF demonstrated a significant association for developing stricture or abscess in our population.

## Discussion

To our knowledge, this is the first study to comprehensively evaluate the association of body composition parameters using a standardized MRE-based semiautomated tool, CrF, and CD complications. Altered body composition parameters and clinical factors such as the age of onset (<40 years), perianal disease, the initial requirement for steroids, early use of anti-inflammatory agents, and smoking history (prior appendectomy) have been suggested as risk factors of complicated CD [8, 10, 11, 14, 31, 32]. Since the duration of CD may last more than 50 years; identifying risk factors for complicated disease over such a long time frame remains extremely difficult [2]. Our data suggest that the occurrence of fistulae is more common in CD patients with a high VAT/SAT ratio or in the presence of CrF.

CD has been traditionally associated with malnutrition and lower BMI [13]. However, the prevalence of obesity in CD patients is increasing [33]. According to a recent metanalysis by Jiang et al. [34] in 2022, obese inflammatory bowel disease patients have an increased risk of surgical complications (OR = 1.45, *p*=<0.001), particularly infectious complications (OR = 1.48, *p* = 0.003) when compared to nonobese patients (including overweight). The impact of obesity on CD behavior has not always been consistent throughout the literature [35]. In our study, BMI did not differ between the groups of inflammatory and complicated disease (*p* = 0.50). 22.8% of our patients had a BMI ≥25 kg/m^2^. Due to the small sample size, patients presenting a BMI higher than 25 kg/m^2^ were not further categorized. Nevertheless, the group of patients with overweight or obesity did not show an increased occurrence of complicated disease (*p* = 0.56). These results are in line with other authors suggesting that BMI alone is not related to disease behavior [10, 33].

Several authors have mentioned that using visceral adiposity as a measure of obesity has more consistently shown an increase in CD complications than using BMI as a marker of obesity [8, 33, 35, 36]. Thiberge et al. [37], in 2018, reported that lower SATI (*p* = 0.009) and VATI (*p* = < 0.001) were inversely correlated with adverse postoperative outcomes in CD patients. In contrast, in our study, TATI, SATI, and VATI did not differ between the groups of complicated and inflammatory disease (*p* = 0.14, *p* = 0.17, and *p* = 0.33, respectively). Our results are in line with Labarthe et al. [15], showing no significant difference in VATI (*p* = 0.34) among CD patients with active compared to inactive disease. The further categorization of our population based on sex-specific cutoff values of SATI (low/normal vs. high SATI) and (low/normal vs. high VATI) did not predict the occurrence of any specific complication (fistulae, abscesses, or strictures). Our data suggest that VAT, VATI, SAT, and SATI alone are inadequate to predict CD complications.

Altered body composition with the development of changed mesenteric adipose tissue is characteristics of CD [35]. The production of tumor necrosis factor α as part of an increased inflammatory response in adipose tissue has been well-documented [38]. According to Kaess et al. [39], VAT or SAT alone provides limited information regarding the relative distribution of body fat when compared to the VAT/SAT ratio. Conelly et al. [33] suggested that the VAT/SAT ratio was also a more reliable predictor of postoperative morbidity in CD patients undergoing an ileocecectomy than BMI (*p* = 0.03). The role of VAT/SAT in CD complications was further explored by Erhayiem et al. [8], who found that the mean VAT/SAT ratio was significantly higher in CD patients with stricturing or fistulizing complications compared to those with uncomplicated disease (*p* = 0.001). In their study, 29 patients with complicated disease were evaluated and the body composition measurements were performed at the L4 level on CT scans, which is not a standard reference location for analyzing body composition parameters [8, 15, 25]. In contrast, our study included a slightly higher amount of patients with complicated disease (*n* = 54), and among penetrating complications alongside fistulae, abscesses were included. The reported high VAT/SAT ratio by Erhayiem et al. was not specific for stricture or fistula. Our data suggest that the use of sex-specific cutoff values for VAT/SAT ratio can be crucial regarding the identification of patients with fistulizing complications (OR: 3.82, 95%, CI 1.34–10.85; *p* = 0.01). Unlike Erhayiem et al., the segmentation of the images in our study was performed using a semiautomated tool on MRE scans at the L3 level, which is a more reproducible tool. In our population, VAT/SAT alone was not associated with a higher recurrence of abscesses (OR: 1.48, 95%, CI 3.14–6.97; *p* = 0.36). The power to detect an association was very limited due to the small number of patients with abscesses (*n* = 7). The differences in body composition parameters within penetrating disease behavior (fistulae and abscesses) remain a topic for further research.

Whether a high VAT/SAT ratio is associated with fistulizing complications is still under debate. In 2015, Büning et al. [36] showed that CD patients with stricturing and fistulizing complications had a high VAT/total fat mass (FM) ratio. In this particular study, VAT was measured with MRI; however, the total FM, with air-displacement plethysmography. Furthermore, only women in clinical remission were included and the administrations of systemic corticosteroid treatment (3 months before the study), severe weight loss (10% of body weight within 6 months before the study), as well as the presence of an ileostomy or colostomy were exclusion criteria. Our population included all CD patients regardless of disease activity, current medication, or prior surgeries, which is more representative of the heterogeneity of the CD population undergoing MRE in clinical routine. Furthermore, the use of air-displacement plethysmography is not a commonly established diagnostic procedure in CD monitoring, which may limit its clinical utility [36].

Bryant et al. [10] suggested that VAT/SAT ratio was associated with stricturing disease behavior (log OR: 1.7; CI, 0.32–3; *p* = 0.01) but not with fistulizing disease. In their study, CD patients between 18 and 50 years were included and VAT/SAT assessment was based on dual-energy X-ray absorptiometry. Furthermore, the classification of complications at baseline was based on clinical data. In contrast, in our study, the definitions of CD-related complications were performed based on the revaluation of the MRE scans, which is a more accurate method to define complicated disease. In our study, a high VAT/SAT was not associated with a higher occurrence of stricturing complications (OR: 0.99, 95%, CI 0.45–2.17; *p* = 0.97). The reason for this is not apparent. Considering that dual-energy X-ray absorptiometry assessment for patients with large VAT values has a poor correlation with MRI and that the gold standard for measuring and analyzing visceral fat comprises MRI and CT our results could not confirm the previous findings of Bryant et al. [10, 40].

CrF has been traditionally associated with small intestinal fibrosis and is characterized by finger-like projections of mesenteric adipose tissue around the inflamed bowel [41]. Data from macroscopic findings indicated that the presence of CrF was associated with hyperplasia of muscularis propia, changes in connective tissue, and ultimately the development of stricture [42]. In a study by Li et al. [43], the degree of CrF assessed by CT was associated with intestinal fibrotic strictures in CD patients (*p* = 0.018). Even so, the association between CrF and stricturing complications is biologically plausible. In our study, the occurrence of CrF in patients with stricturing complications when compared to patients without stricturing complications was not significantly different (*p* = 0.18). The reason for this is not apparent. It has been reported that connective tissue changes including CrF are related to local effects of underlying chronic inflammation [42]. In our study, as well as in previous studies, no distinction between inflammatory and fibrotic stenotic changes was made, being presumably the latter stronger related to adipose tissue changes including CrF [44].

Althoff et al. [45] identified that CrF evaluated by MRI was associated with a complicated course of abdominal surgery in CD patients. In our study, CrF tended to be more common in patients with complicated compared to inflammatory disease (*p* = 0.11). In our population, fistulizing complications rather than stricturing complications were more likely to occur in patients with CrF (OR 5.07, 95% CI 1.76–14.56; *p*= <0.001). The observation that CrF was common in patients with fistulae is plausible since CrF has been described as a protective response where mesenteric adipose tissue migrates to sites of gut barrier dysfunction to prevent systemic dissemination of potentially harmful bacterial antigens that have translocated across the barrier from the gut lumen [9]. Furthermore, some bacteria might directly infect endothelial cells and adipocytes, causing them to proliferate, ultimately generating the development of new vessels and CrF of the mesentery [9, 46]. As a result, the walls of fistulae might result from neoangiogenesis or lymphangiogenesis that occurs in the bowel wall [46].

The incidence of sarcopenia in our population was 59.6%. This is higher compared with the previous incidence reported by Thiberge et al. [37] (33.6%) and comparable with the incidence in CD patients reported by Labarthe et al. [15] (50%). In a meta-analysis by Erős et al. [47], in 2020, sarcopenia was identified as an independent predictor for rate of surgery (OR = 1.826; 95% CI 0.913–3.654; *p* = 0.089). In our study, sarcopenia was not associated with the occurrence of complicated disease. Even though both groups were well-matched for current drug therapy, this clinical information was available for only 86.8% of the patients. As it is well known that glucocorticoids induce muscle atrophy, we cannot exclude that these results were influenced by the current CD-related therapies [48].

As described by Labarthe et al. [15] MRI measurements of body composition parameters are feasible and reproducible, particularly with the help of semiautomated methods. MRE has a higher accuracy in detecting CrF and fistula than CT; the avoidance of radiation exposure is also an advantage [23]. The clinical perspective offered by our data suggests that the MRE-based stratification of CD patients in high VAT/SAT group, as well as the radiological assessment of CrF, should be recognized as a new potential prognostic factor for the occurrence of fistulizing complications. Our results should generate further studies, particularly focusing on the quantitative assessment of CrF in MRE. Ultimately the clinical utility and setting (assessment at diagnosis, monitoring symptomatic or asymptomatic patients, or postoperative follow-up) must be determined in prospective multicentric studies. In agreement with Xiong et al. [49], we consider that differences at the L3 and L5 levels, in addition to the L3 level, should be studied when analyzing body composition parameters in CD patients, particularly by assessing adipose tissue changes. The impact of body composition changes over time on disease behavior also requires further investigation.

Our study has some limitations, CD patients with contraindications for MRE, such as electrically, magnetically, or mechanically activated devices or known adverse reactions to gadolinium contrast media, were not included in our study [21]. In our study, the anthropometric data were obtained from clinical records, which could not be verified. Additionally, the quantification of body composition parameters from MRE scans has not been completely standardized, and there is wide variability concerning protocol optimization. The retrospective methodology of the study did not allow a rigorous evaluation of body composition changes over time or the consideration of the role of surgery and current drug therapies as additional factors to predict complicated disease. CD patients with acute intestinal complications like low intestinal bleeding, perforation, and intestinal obstruction were not included. Besides, in acute settings at our institution, most of the CD patients presenting an abscess undergo a CT scan. The small sample size of patients presenting penetrating complications, particularly abscesses is another limitation of our study.

## Conclusions

Body composition measurements in CD patients displayed no statistically significant difference between the groups of inflammatory and complicated disease. Nonetheless, CD patients stratified in the group of high VAT/SAT ratio and the presence of CrF should be recognized as risk groups for the occurrence of fistulae.

## Statement of Ethics

This study was conducted according to the principles of the Declaration of Helsinki. This study was approved by the Institutional Review Board Ethics Committee (Number: 145/21), Otto-von-Guericke University, Magdeburg, Germany. For this retrospective study, the requirement of informed consent was waived.

## Conflict of Interest Statement

The authors have no conflict of interest to declare.

## Funding Sources

There was no financial support for this study.

## Author Contributions

AS and FB conceived and designed the study; FB, OM, MT, and BM contributed to the collection of the clinical data and BM performed the segmentation of the MRE scans. AS and BM evaluated the MRE scans. FB and PR contributed to the manuscript writing. FB and RD contributed to the statistical analysis. MP, JO, and PR contributed to the critical revision of the manuscript. All authors approved the final manuscript for publication.

## Data Availability Statement

All data generated or analyzed during this study are included in this article and its online supplementary material files. Further inquiries can be directed to the corresponding author (FB).

## Supplementary Material

Supplementary data

## Figures and Tables

**Table 1:** Baseline characteristics of all CD patients (*n* = 114)

	All CD patients, (*n* = 114), *n* (%)	Inflammatory disease (*n* = 54), *n* (%)	Complicated disease, (*n* = 60), *n* (%)	*p* value
Male	60 (52.6)	30 (55.6)	30 (50.0)	0.55
Age at baseline (years), median, [IQR]	35.50 [27.0–46.3]	38.50 [26.8–47.3]	33.00 [27.0–45.0]	0.91
Age at diagnosis (years), median, [IQR]	26.00 [17.0–36.0]	28.00 [17.0–35.3]	25.50 [17.0–36.8]	0.84
Age of onset				
A1	24 (21.1)	11 (20.4)	13 (21.7)	0.87
A2	69 (60.5)	35 (64.8)	34 (56.7)	0.37
A3	21 (18.4)	8 (14.8)	13 (21.7)	0.35
Current drug therapy^a^				
Corticosteroids	36 (31.6)	19 (35.9)	17 (28.3)	0.27
Biological therapy	22 (19.3)	11 (20.4)	11 (18.3)	0.63
Immunomodulator	25 (21.9)	14 (25.9)	11 (18.3)	0.22
5-aminosalicylic acid	14 (12.3)	5 (9.2)	9 (15.0)	0.41
Current smoker	13 (11.4)	6 (11.1)	7 (11.7)	0.93
CRP (<5 ng/mL)	88 (77.2)	40 (74.1)	48 (80.0)	0.45
BMI (kg/m^2^), median, [IQR]	21.94 [19.4–24.9]	21.32 [18.8–35.3]	22.21 [19.5–25.2]	0.50
BMI <18.5 kg/m^2^	20 (17.5)	11 (20.4)	9 (15.0)	0.45
BMI 18.5-24.9 kg/m^2^	68 (59.6)	32 (59.3)	36 (60.0)	0.94
BMI >25 kg/m^2^	26 (22.8)	11 (20.4)	15 (25.0)	0.56

Continuous variables are reported as median and interquartile range (IQR). CD, Crohn's disease; CRP, C-reactive, protein; BMI, body mass index.

a Current therapy information was available for 99 patients only.

**Table 2:** Body composition parameters in the groups of inflammatory and complicated CD (*n* = 114)

Parameter	Inflammatory disease, total (*n* = 54)	Complicated disease total *(n* = 60)	*p* value
TAT (cm^2^), median [IQR]	159.45 [110.2–250.6]	196.45 [126.6–287.5]	0.17
TATI (cm^2^/m^2^), median [IQR]	57.23 [36.5–85.8]	69.23 [42.9–98.2]	0.14
SAT (cm^2^), median [IQR]	92.75 [46.2–139.4]	109.40 [63.0–184.0]	0.22
SATI (cm^2^/m^2^), median [IQR]	30.75 [14.4–50.7]	36.20 [20.0–61.1]	0.17
VAT (cm^2^), median [IQR]	59.55 [38.0–95.5]	69.45 [45.9–96.6]	0.37
VATI (cm^2^/m^2^), median [IQR]	21.44 [13.3–34.5]	26.87 [15.2–35.1]	0.33
VAT/SAT ratio, median [IQR]	0.70 [0.4–1.1]	0.70 [0.4–1.2]	0.77
IMAT (cm^2^), median [IQR]	3.85 [1.9–8.3]	3.95 [2.4–4.0]	0.64
SMT (cm^2^), M ± SD	111.14±36.57	117.99±36.35	0.32
SMI (cm^2^/m^2^), M ± SD	37.30±10.39	39.67±10.18	0.22

Continuous variables are reported as mean (M) ± standard deviation (SD), or median and interquartile range (IQR). TAT, total adipose tissue; TATI, total adipose tissue index; SAT, subcutaneous adipose tissue; SATI, subcutaneous adipose tissue index; VAT, visceral adipose tissue; VATI, visceral adipose tissue index; IMAT, intramuscular adipose tissue; SMT, skeletal muscle tissue; SMI, skeletal muscle index; M, mean; SD, standard deviation.

**Table 3:** Body composition groups based on sex-specific values, creeping fat, and occurrence of complicated disease (*n* = 114)

Groups	Patients with inflammatory disease, total (*n* = 54), *n* (%)	Patients with Complicated disease total *(n* = 60), *n* (%)	*p* value
Low/normal SATI	31 (27.2)	28 (24.6)	0.25
High SATI	23 (20.2)	32 (28.1)	
Low/normal VATI	44 (38.6)	48 (42.1)	0.84
High VATI	10 (8.8)	12 (10.5)	
Low/normal VAT/SAT ratio	37 (32.5)	38 (33.3)	0.56
High VAT/SAT ratio	17(14.9)	22 (19.3)	
Non-sarcopenic	20 (17.5)	26 (22.8)	0.50
Sarcopenic	34 (29.8)	34 (29.8)	
CrF absent	44 (38.5)	41 (35.9)	0.11
CrF present	10 (8.7)	19 (16.6)	

SATI, subcutaneous adipose tissue index; VATI, visceral adipose tissue index; VAT/SAT ratio, visceral-to-subcutaneous fat ratio; CrF, creeping fat.

**Table 4:** Body composition groups, creeping fat, and occurrence of fistulae (*n* = 114)

Groups	Patients without fistula, total (*n* = 96), *n* (%)	Patients with fistula, total (*n* = 18), *n* (%)	*p* value
Low/normal SATI	48 (50)	11 (61.1)	0.39
High SATI	48 (50)	7 (38.9)	
Low/normal VATI	77 (80.2)	15 (83.3)	1.00
High VATI	19 (19.8)	3 (16.7)	
Low/normal VAT/SAT ratio	68 (70.8)	7 (38.9)	0.01
High VAT/SAT ratio	28 (29.2)	11 (61.1)	
Non-sarcopenic	40 (41.7)	6 (33.3)	0.51
Sarcopenic	56 (58.3)	12 (66.7)	
CrF absent	77 (80.2)	8 (44.4)	<0.001
CrF present	19 (19.8)	10 (55.6)	

SATI, subcutaneous adipose tissue index; VATI, visceral adipose tissue index; VAT/SAT ratio, visceral-to-subcutaneous fat ratio; CrF, creeping fat.

**Table 5:** OR and 95% CI for the occurrence of fistula (*n* = 114)

Groups	OR	95% CI	*p* value
High SATI versus low/normal SATI	0.64	0.28–1.78	0.86
High VATI versus low/normal VATI	0.81	0.23–3.09	0.76
High VAT/SAT versus low/normal VAT/SAT	3.82	1.34–10.85	0.01
Sarcopenic versus non-sarcopenic	1.43	0.50–4.13	0.51
CrF present versus CrF absent	5.07	1.76–14.56	<0.001

SATI, subcutaneous adipose tissue index; VATI, visceral adipose tissue index; VAT/SAT, visceral-to-subcutaneous fat ratio; CrF, creeping fat; OR, odds ratio; CI, confidence intervals.

**Table 6:** OR and 95% CI for the occurrence of stricture (*n* = 114)

Groups	OR	95% CI	*p* value
High SATI versus low/normal SATI	1.21	0.57–2.56	0.50
High VATI versus low/normal VATI	0.78	0.30–2.03	0.61
High VAT/SAT versus low/normal VAT/SAT	0.99	0.45–2.17	0.97
Sarcopenic versus non-sarcopenic	0.63	0-30–1.36	0.24
CrF present versus CrF absent	1.77	0.76–4.15	0.19

SATI, subcutaneous adipose tissue index; VATI, visceral adipose tissue index; VAT/SAT, visceral-to-subcutaneous fat ratio; CrF, creeping fat.

## References

[1] Zhao M, Gönczi L, Lakatos PL, Burisch J (2021). The burden of inflammatory bowel disease in europe in 2020. J Crohn's Colitis.

[2] Cosnes J, Bourrier A, Nion-Larmurier I, Sokol H, Beaugerie L, Seksik P (2012). Factors affecting outcomes in Crohn's disease over 15 years. Gut.

[3] Satsangi J, Silverberg MS, Vermeire S, Colombel JF (2006). The Montreal classification of inflammatory bowel disease controversies, consensus, and implications. Gut.

[4] Rozendorn N, Amitai MM, Eliakim RA, Kopylov U, Klang E (2018). A review of magnetic resonance enterography-based indices for quantification of Crohn's disease inflammation. Therap Adv Gastroenterol.

[5] Schütz L, Radke M, Menzel S, Däbritz J (2019). Long-term implications of structured transition of adolescents with inflammatory bowel disease into adult health care a retrospective study. BMC Gastroenterol.

[6] Irwin J, Ferguson E, Simms LA, Hanigan K, Carbonnel F, Radford-Smith G (2017). A rolling phenotype in Crohn's disease. PLoS One.

[7] GBD 2017 Inflammatory Bowel Disease Collaborators, Sepanlou SG, Ikuta K, Vahedi H, Bisignano C, Safiri S (2020). The global and national burden of inflammatory bowel disease in 195 countries and territories,1990–2017 a systematic analysis for the Global Burden of Disease Study 2017. Lancet Gastroenterol Hepatol.

[8] Erhayiem B, Dhingsa R, Hawkey CJ, Subramanian V (2011). Ratio of visceral to subcutaneous fat area is a biomarker of complicated crohn's disease. Clin Gastroenterol Hepatol.

[9] Ha CWY, Martin A, Sepich-Poore GD, Shi B, Wang Y, Gouin K (2020). Translocation of viable gut microbiota to mesenteric adipose drives formation of creeping fat in humans. Cell.

[10] Bryant RV, Schultz CG, Ooi S, Goess C, Costello SP, Vincent AD (2019). Visceral adipose tissue is associated with stricturing crohn's disease behavior fecal calprotectin and quality of life. Inflamm Bowel Dis.

[11] Dhaliwal A, Quinlan JI, Overthrow K, Greig C, Lord JM, Armstrong MJ (2021). Sarcopenia in inflammatory bowel disease a narrative overview. Nutrients.

[12] Cruz-Jentoft AJ, Baeyens JP, Bauer JM, Boirie Y, Cederholm T, Landi F (2010). Sarcopenia European consensus on definition and diagnosis: report of the European working group on sarcopenia in older people. Age Ageing.

[13] Grillot J, D'Engremont C, Parmentier AL, Lakkis Z, Piton G, Cazaux D (2020). Sarcopenia and visceral obesity assessed by computed tomography are associated with adverse outcomes in patients with Crohn's disease. Clin Nutr.

[14] Zhou Z, Xiong Z, Xie Q, Xiao P, Zhang Q, Gu J (2021). Computed tomography-based multiple body composition parameters predict outcomes in Crohn's disease. Insights Imaging.

[15] Labarthe G, Dolores M, Verdalle-Cazes M, Charpentier C, Roullee P, Dacher JN (2020). Magnetic resonance imaging assessment of body composition parameters in Crohn's disease. Dig Liver Dis.

[16] Ahmad R, Ajlan AM, Eskander AA, Alhazmi TA, Khashoggi K, Wazzan MA (2021). Magnetic resonance imaging in the management of Crohn's disease a systematic review and meta-analysis. Insights Imaging.

[17] Manetta R, Capretti I, Belleggia N, Marsecano C, Viscido A, Bruno F (2019). Magnetic resonance enterography (MRE) and ultrasonography (US) in the study of the small bowel in crohn's disease state of the art and review of the literature. Acta Biomed.

[18] Stoddard PB, Ghazi LJ, Wong-You-Cheong J, Cross RK, Vandermeer FQ (2015). Magnetic resonance enterography state of the art. Inflamm Bowel Dis.

[19] Maaser C, Sturm A, Vavricka SR, Kucharzik T, Fiorino G, Annese V (2019). ECCO-ESGAR Guideline for Diagnostic Assessment in IBD Part 1 initial diagnosis, monitoring of known IBD, detection of complications. J Crohns Colitis.

[20] Stange EF, Travis SPL, Vermeire S, Beglinger C, Kupcinkas L, Geboes K (2006). European evidence based consensus on the diagnosis and management of Crohn's disease definitions and diagnosis. Gut.

[21] Barat M, Hoeffel C, Bouquot M, Jannot AS, Dautry R, Boudiaf M (2019). Preoperative evaluation of small bowel complications in Crohn's disease comparison of diffusion-weighted and contrast-enhanced MR imaging. Eur Radiol.

[22] Cravo ML, Velho S, Torres J, Costa Santos MP, Palmela C, Cruz R (2017). Lower skeletal muscle attenuation and high visceral fat index are associated with complicated disease in patients with Crohn's disease an exploratory study. Clin Nutr ESPEN.

[23] Amitai MM, Raviv-Zilka L, Hertz M, Erlich Z, Konen E, Ben-Horin S (2015). Main imaging features of crohn's disease agreement between MR-enterography and CT-enterography. Isr Med Assoc J.

[24] Park J, Gil JR, Shin Y, Won SE, Huh J, You MW (2019). Reliable and robust method for abdominal muscle mass quantification using CT/MRI an explorative study in healthy subjects. PLoS One.

[25] Koitka S, Kroll L, Malamutmann E, Oezcelik A, Nensa F (2021). Fully automated body composition analysis in routine CT imaging using 3D semantic segmentation convolutional neural networks. Eur Radiol.

[26] Feng Z, Rong P, Luo M, Sun X, Wang W (2019). Influence of methods used to establish sarcopenia cutoff values for skeletal muscle measures using unenhanced and contrast-enhanced computed tomography images. JPEN J Parenter Enteral Nutr.

[27] Magro DO, Barreto MRL, Cazzo E, Camargo MG, Kotze PG, Coy CSR (2018). Visceral fat is increased in individuals with Crohn's disease a comparative analysis with healthy controls. Arq Gastroenterol.

[28] Martin L, Birdsell L, MacDonald N, Reiman T, Clandinin MT, McCargar LJ (2013). Cancer cachexia in the age of obesity skeletal muscle depletion is a powerful prognostic factor, independent of body mass index. J Clin Oncol.

[29] Kobayashi T, Kawai H, Nakano O, Abe S, Kamimura H, Sakamaki A (2018). Prognostic value of subcutaneous adipose tissue volume in hepatocellular carcinoma treated with transcatheter intra-arterial therapy. Cancer Manag Res.

[30] Hamaguchi Y, Kaido T, Okumura S, Kobayashi A, Shirai H, Yao S (2020). Including body composition in MELD scores improves mortality prediction among patients awaiting liver transplantation. Clin Nutr.

[31] Beaugerie L, Seksik P, Nion-Larmurier I, Gendre JP, Cosnes J (2006). Predictors of crohn's disease. Gastroenterology.

[32] Yoo JH, Holubar S, Rieder F (2020). Fibrostenotic strictures in Crohn's disease. Intest Res.

[33] Connelly TM, Juza RM, Sangster W, Sehgal R, Tappouni RF, Messaris E (2014). Volumetric fat ratio and not body mass index is predictive of ileocolectomy outcomes in Crohn's disease patients. Dig Surg.

[34] Jiang K, Chen B, Lou D, Zhang M, Shi Y, Dai W (2022). Systematic review and meta-analysis association between obesity/overweight and surgical complications in IBD. Int J Colorectal Dis.

[35] Bilski J, Mazur-Bialy A, Wojcik D, Surmiak M, Magierowski M, Sliwowski Z (2019). Role of obesity mesenteric adipose tissue and adipokines in inflammatory bowel diseases. Biomolecules.

[36] Büning C, Von Kraft C, Hermsdorf M, Gentz E, Wirth EK, Valentini L (2015). Visceral adipose tissue in patients with Crohn's disease correlates with disease activity inflammatory markers and outcome. Inflamm Bowel Dis.

[37] Thiberge C, Charpentier C, Gillibert A, Modzelewski R, Dacher JN, Savoye G (2018). Lower subcutaneous or visceral adiposity assessed by abdominal computed tomography could predict adverse outcome in patients with Crohn's disease. J Crohn's Colitis.

[38] Hass DJ, Brensinger CM, Lewis JD, Lichtenstein GR (2006). The impact of increased body mass index on the clinical course of crohn's disease. Clin Gastroenterol Hepatol.

[39] Kaess BM, Pedley A, Massaro JM, Murabito J, Hoffmann U, Fox CS (2012). The ratio of visceral to subcutaneous fat a metric of body fat distribution is a unique correlate of cardiometabolic risk. Diabetologia.

[40] Murata H, Yagi T, Midorikawa T, Torii S, Takai E, Taguchi M (2022). Comparison between DXA and MRI for the visceral fat assessment in athletes. Int J Sports Med.

[41] Dickson I (2020). Creeping fat in Crohn's disease explained. Nat Rev Gastroenterol Hepatol.

[42] Mao R, Kurada S, Gordon IO, Baker ME, Gandhi N, McDonald C (2019). The mesenteric fat and intestinal muscle interface creeping fat influencing stricture formation in crohn's disease. Inflamm Bowel Dis.

[43] Li XH, Feng ST, Cao QH, Coffey JC, Baker ME, Huang L (2021). Degree of creeping fat assessed by computed tomography enterography is associated with intestinal fibrotic stricture in patients with crohn's disease a potentially novel mesenteric creeping fat index. J Crohns Colitis.

[44] Zhu J, Zhang F, Liu F, He W, Tian J, Han H (2015). Identifying the inflammatory and fibrotic bowel stricture MRI diffusion-weighted imaging in Crohn's disease. Radiol Infect Dis.

[45] Althoff P, Schmiegel W, Lang G, Nicolas V, Brechmann T (2019). Creeping fat assessed by small bowel MRI is linked to bowel damage and abdominal surgery in crohn's disease. Dig Dis Sci.

[46] Pierce ES (2009). Where are all the Mycobacterium avium subspecies paratuberculosis in patients with crohn's disease?. PLoS Pathog.

[47] Erős A, Soós A, Hegyi P, Szakács Z, Benke M, Szűcs Á (2020). Sarcopenia as an independent predictor of the surgical outcomes of patients with inflammatory bowel disease a meta-analysis. Surg Today.

[48] Sato AY, Richardson D, Cregor M, Davis HM, Au ED, McAndrews K (2017). Glucocorticoids induce bone and muscle atrophy by tissue-specific mechanisms upstream of E3 ubiquitin ligases. Endocrinology.

[49] Xiong Z, Zhou Z, Hao L, Li Y, Hu X, Hu D (2022). The relationship between perianal fistula activity and abdominal adipose tissue in Crohn's disease an observational study. Insights Imaging.

