# Supplementary material for: Body Composition Predictors of Complicated Crohn's Disease
Source: Dig Dis. 2023 Jan 31;41(4):589–99. doi: 10.1159/000529426 (PMC10777712; doi:10.1159/000529426)
Supplement: Supplementary file 1 — Supplementary data [file ddi-0041-0589-s01.docx]

Supplementary material

| Suppl. Table 1. MRI sequences of MRE protocol | | | |  |
| --- | --- | --- | --- | --- |
| Sequences | Plane | Slice thickness (mm) | FOV (mm) | TR/TE (ms) |
| Precontrast media | | | | |
| T2 SSH-TSE | coronal | 5 | 450x450 | 599/80 |
| T2 SPAIR | coronal | 5 | 450x450 | 605/80 |
| b-FFE | coronal | 6 | 410x410 | 6/3 |
| T1 TFE | axial | 6 | 355x355 | 216/5 |
| THRIVE | axial | 6 | 355x355 | 4/2 |
| After contrast medium | | | | |
| THRIVE | coronal | 4 | 405x405 | 6/3 |
| T2 SPAIR | axial | 6 | 375x375 | 1200/60 |
| T2 SSH-TSE | axial | 6 | 375x375 | 1200/80 |
| T1 WATS | axial | 6 | 375x375 | 227/5 |
| THRIVE | axial | 6 | 355x355 | 4/2 |
| DWI (b0 and b 600) | axial | 6 | 340x340 | 1637/61 |
| MRI, magnetic resonance imaging; MRE, magnetic resonance imaging enterography; FOV, field of view; TR, time repetition; TE, time echo; SSH-TSE, single-shot turbo spin echo; SPIAR, spectral attenuated inversion recovery; b-FFE, balanced Fast Field Echo; TFE, Turbo Field Echo; THRIVE, T1-weighted High-Resolution Isotropic Volume Examination; T1 WATS, T1 gradient echo with water selective excitation; DWI, diffusion-weighted magnetic resonance Imaging. | | | | |

| Suppl. Table 2a. Body composition groups, creeping fat, and occurrence of abscesses (*n*=114) | | | |
| --- | --- | --- | --- |
| Groups | Patients without abscess, total (*n*=107), n (%) | Patients with Abscess, total (*n* =7), n (%) | *p-*value |
| Low/normal SATI | 56 (52.3) | 3 (42.9) | 0.71 |
| High SATI | 51 (47.7) | 4 (57.1) |  |
|  |  |  |  |
| Low/normal VATI | 86 (80.4) | 6 (85.7) | 1.00 |
| High VATI | 21 (19.6) | 1 (14.3) |  |
|  |  |  |  |
| Low/normal VAT/SAT ratio | 71 (66.4) | 4 (57.1) | 0.62 |
| High VAT/SAT ratio | 36 (33.6) | 3 (42.9) |  |
|  |  |  |  |
| Non-sarcopenic | 44 (41.1) | 2 (28.6) | 0.70 |
| Sarcopenic | 63 (58.9) | 5 (71.4) |  |
|  |  |  |  |
| Without CrF | 81 (75.7) | 4 (57.1) | 0.37 |
| With CrF | 26 (24.3) | 3 (42.9) |  |
| SATI, subcutaneous adipose tissue index; VATI, visceral adipose tissue index; VAT/SAT ratio, visceral-to-subcutaneous fat ratio; CrF, creeping fat. | | | |

| Suppl. Table 2b. Body composition groups, creeping fat, and occurrence of stricturing complications (*n*=114) | | | |
| --- | --- | --- | --- |
| Groups | Patients without stricture, total (*n*=67), n (%) | Patients with stricture, total (*n*=47), n (%) | *p-*value |
| Low/normal SATI | 36 (64.2) | 23 (48.9) | 0.61 |
| High SATI | 31 (46.3) | 24 (51.1) |  |
|  |  |  |  |
| Low/normal VATI | 53 (79.1) | 39 (83.0) | 0.61 |
| High VATI | 14 (20.9) | 8 (17.0) |  |
|  |  |  |  |
| Low/normal VAT/SAT ratio | 44 (65.7) | 31 (66.0) | 0.98 |
| High VAT/SAT ratio | 23 (34.3) | 16 (34.0) |  |
|  |  |  |  |
| Non-sarcopenic | 24 (35.8) | 22 (46.8) | 0.24 |
| Sarcopenic | 43 (64.2) | 25 (53.2) |  |
|  |  |  |  |
| CrF absent | 53 (79.1) | 32 (68.1) | 0.18 |
| CrF present | 14 (20.9) | 15 (31.9) |  |
| SATI subcutaneous adipose tissue index; VATI, visceral adipose tissue index; VAT/SAT ratio, visceral-to-subcutaneous fat ratio, CrF, creeping fat. | | | |

| Suppl. Table 3. Odds ratio (OR) and 95% confidence intervals (CI) for the occurrence of abscess (*n*=114) | | | |
| --- | --- | --- | --- |
| Groups | OR | 95%CI | *p-*value |
|  |  |  |  |
| High SATI vs low/normal SATI | 1.46 | 0.31 – 6.86 | 0.63 |
| High VATI vs. low/normal VATI | 0.683 | 0.08 – 5.98 | 0.73 |
| High VAT/SAT vs. low/normal VAT/SAT | 1.48 | 3.14 – 6.97 | 0.36 |
| Sarcopenic vs. non-sarcopenic | 1.75 | 0.32 – 9.41 | 0.51 |
| CrF present vs. CrF absent | 2.34 | 0.49 – 11.13 | 0.29 |
| SATI, subcutaneous adipose tissue index; VATI, visceral adipose tissue index; VAT/SAT, visceral-to-subcutaneous fat ratio; CrF, creeping fat. | | | |
